# Registros Clínicos no Infarto Agudo do Miocárdio

**DOI:** 10.36660/abc.20230066

**Published:** 2023-08-10

**Authors:** Miguel Alejandro Rodriguez-Ramos

**Affiliations:** 1 Hospital Provincial Universitario Camilo Cienfuegos Sancti Spiritus Cuba Hospital Provincial Universitario Camilo Cienfuegos – Cardiology, Sancti Spiritus – Cuba

**Keywords:** Registros Médicos, Infarto Miocárdio, Países em Desenvolvimento

**Caro editor**

Aprecio os esforços de Alves et al.^1^ quando tentaram avaliar a mortalidade hospitalar por todas as causas em pacientes internados por STEMI e NSTEMI em hospitais na América Latina e no Caribe a partir de 2000, concluindo que a mortalidade hospitalar em países de baixa e média renda era alta em comparação com as taxas relatadas em países de alta renda. Para melhorar essas estimativas, se deve buscar maior uso da terapia de reperfusão.

Efetivamente, a qualidade do atendimento ao infarto agudo do miocárdio (IAM) na América Latina está abaixo dos padrões dos países desenvolvidos. Isso se deve a vários motivos: falta de recursos, treinamento inadequado dos profissionais de saúde e infraestrutura precária. Ainda assim, alguns esforços estão sendo feitos para melhorar a qualidade da assistência ao IAM.

Alguns países implementaram programas para treinar profissionais de saúde e fornecer-lhes os equipamentos e medicamentos necessários para diagnosticar e tratar o IAM. Além disso, alguns governos estão trabalhando para melhorar o acesso à saúde aumentando o financiamento e expandindo a cobertura de seguro. Finalmente, alguns têm implementado registros de pacientes com Infarto Agudo do Miocárdio. Esses registros de infarto do miocárdio desempenham um papel vital na compreensão da carga dessa doença em cenários de renda média, pois essa é uma das principais causas de morte em todo o mundo. Dados precisos sobre a incidência, fatores de risco e resultados da doença são essenciais para o desenvolvimento de estratégias eficazes de prevenção e tratamento.

Os registros coletam dados de pacientes com diagnóstico de infarto do miocárdio, incluindo informações demográficas, histórico médico e detalhes do evento de infarto do miocárdio. Esses dados podem ser usados para entender a epidemiologia da doença, incluindo a incidência e prevalência de infarto do miocárdio, fatores de risco e resultados. Em países de renda média, essas informações podem ajudar a orientar a alocação de recursos e o desenvolvimento de estratégias direcionadas de prevenção e tratamento. Além disso, se uma determinada opção de tratamento parece mais eficaz do que outras em uma determinada população de pacientes, os profissionais de saúde podem usar essas informações para tomar decisões de tratamento mais informadas para futuros pacientes. Isso pode levar a melhores resultados para os pacientes e melhorar a qualidade do atendimento.

Um aspecto importante dos registros de IM em cenários de renda média é a capacidade de identificar disparidades na carga de doença. Se os dados do registro mostrarem que certas populações de pacientes não estão recebendo o mesmo nível de atendimento que outras, os profissionais de saúde podem trabalhar para abordar essas disparidades e melhorar o atendimento a esses pacientes.

Essas informações podem ajudar políticas e programas que abordam essas disparidades. Os registros podem ser usados para avaliar o impacto de intervenções como programas de cessação do tabagismo, programas de controle da hipertensão e terapia com estatinas.

Além disso, os registros de IM também podem ser usados para identificar e rastrear pacientes com alto risco de um IM subsequente. Ao identificar esses pacientes, os profissionais de saúde podem tomar medidas para reduzir o risco e prevenir outro ataque cardíaco. Os registros de infarto do miocárdio também desempenham um papel crítico na pesquisa e no desenvolvimento de novos tratamentos. Ao fornecer um grande conjunto de dados de pacientes e seus resultados, os pesquisadores podem entender melhor as causas subjacentes dos ataques cardíacos e desenvolver novos tratamentos para melhorar os resultados dos pacientes.

Em geral, os registros de infarto do miocárdio são uma ferramenta vital para melhorar a qualidade do atendimento a pacientes que sofreram um ataque cardíaco. Ao coletar dados demográficos do paciente, fatores de risco, resultados de testes diagnósticos, opções de tratamento e resultados, os profissionais de saúde podem identificar padrões e tendências que podem ajudá-los a melhorar o atendimento prestado a pacientes com infarto do miocárdio.

Assim, o American College of Cardiology lançou a *Global Heart Attack Treatment Initiative* (GHATI)^2^ para melhorar o tratamento e os resultados de pacientes com infarto agudo do miocárdio em todo o mundo. A iniciativa se concentra em promover o uso de diretrizes baseadas em evidências e melhores práticas para o tratamento de pacientes com ataque cardíaco, bem como aumentar a conscientização e a educação sobre a importância do tratamento oportuno e eficaz. O GHATI também trabalha para facilitar a colaboração internacional e a troca de informações e recursos para melhorar o tratamento de ataques cardíacos globalmente.

Apesar de ter sido lançado há vários anos, nenhum relatório atual sobre os dados do paciente está incluído neste registro. Aparentemente, o surgimento do COVID-19 pode ter influenciado o atraso nas publicações deste projeto.

Para concluir, os registros de IM são essenciais para entender a carga de doença em cenários de renda média. Eles fornecem informações valiosas sobre a incidência, fatores de risco e resultados do infarto do miocárdio e podem ser usados para identificar disparidades na carga da doença e monitorar a eficácia das estratégias de prevenção e tratamento. Esta informação é essencial para o desenvolvimento de abordagens eficazes para reduzir a carga de IM em países de renda média.

## References

[1] Alves L, Ziegelmann PK, Ribeiro V, Polanczyk C (2022). Hospital Mortality from Myocardial Infarction in Latin America and the Caribbean: Systematic Review and Meta-Analysis. Arq Bras Cardiol.

[2] Levenson B, Herrera C, Wilson BH (2020). New ACC Global Heart Attack Treatment Initiative: Improving STEMI Care Worldwide. J Am Coll Cardiol.

