# Supplementary material for: Registros Clínicos no Infarto Agudo do Miocárdio
Source: Arq Bras Cardiol. 2023 Aug 10;120(8):e20230066. [Article in Portuguese] doi: 10.36660/abc.20230066 (PMC10519247; doi:10.36660/abc.20230066)
Supplement: Translation

# Translation: Clinical registries in Acute Myocardial Infarction

**Authors:** Miguel Alejandro Rodriguez-Ramos

**Affiliations:** 1 Cuba Hospital Provincial Universitario Camilo Cienfuegos – Cardiology, Sancti Spiritus – Cuba

**Keywords:** Medical Records, Myocardial Infarction, Developing Countries

**Dear Editor**

I appreciate the efforts of Alves et al.^1^when they tried to evaluate all-cause in-hospital mortality in patients admitted for STEMI and NSTEMI in hospitals in Latin America and the Caribbean from 2000 onward, concluding that in-hospital mortality in low- and middle-income countries was high in comparison with rates reported in high-income countries and to improve these estimates, higher use of reperfusion therapy must be pursued.

Effectively, the quality of care for AMI in Latin America is below the standards of developed countries. This is due to several reasons: lack of resources, inadequate training of healthcare professionals, and poor infrastructure. Still, some efforts are being made to improve the quality of care for AMI.

Some countries have implemented programs to train healthcare professionals and provide them with the necessary equipment and medications to diagnose and treat AMI. Additionally, some governments are working to improve access to healthcare by increasing funding and expanding insurance coverage. Finally, some have implemented registries of patients with Acute Myocardial Infarction. These registries of myocardial infarction play a vital role in understanding the burden of this disease in middle-income scenarios, as this is a leading cause of death worldwide. Accurate data on the incidence, risk factors, and disease outcomes are essential for developing effective prevention and treatment strategies.

Registries collect data on patients diagnosed with MI, including demographic information, medical history, and details of the MI event. This data can be used to understand the epidemiology of the disease, including the incidence and prevalence of MI, risk factors, and outcomes. In middle-income countries, this information can help to guide the allocation of resources and the development of targeted prevention and treatment strategies. Moreover, if a particular treatment option appears more effective than others in a particular patient population, healthcare providers can use this information to make more informed treatment decisions for future patients. This can lead to better patient outcomes and improved quality of care.

One important aspect of MI registries in middle-income scenarios is the ability to identify disparities in disease burden. If data from the registry shows that certain patient populations are not receiving the same level of care as others, healthcare providers can work to address these disparities and improve care for these patients.

This information can help to inform policies and programs that address these disparities. Registries can be used to evaluate the impact of interventions such as smoking cessation programs, hypertension management programs, and statin therapy.

In addition, MI registries can also be used to identify and track patients who are at high risk for a subsequent MI. By identifying these patients, healthcare providers can take steps to reduce their risk and prevent another heart attack. Registries of myocardial infarction also play a critical role in research and developing new treatments. By providing a large dataset of patients and their outcomes, researchers can better understand the underlying causes of heart attacks and develop new treatments to improve patient outcomes.

Overall, registries of myocardial infarction are a vital tool for improving the quality of care for patients who have experienced a heart attack. By collecting data on patient demographics, risk factors, diagnostic test results, treatment options, and outcomes, healthcare providers can identify patterns and trends that can help them improve the care they provide to patients with MI.

Accordingly, The American College of Cardiology launched the Global Heart Attack Treatment Initiative (GHATI)^2^to improve the treatment and outcomes of patients with acute myocardial infarction worldwide. The initiative focuses on promoting the use of evidence-based guidelines and best practices for managing heart attack patients, as well as increasing awareness and education about the importance of timely and effective treatment. GHATI also works to facilitate international collaboration and exchange of information and resources to improve heart attack care globally.

Despite being launched several years ago, no current report on patient data is included in this registry. Apparently, the COVID-19 emergence may have influenced the delay in the publications of this project.

To conclude, MI registries are critical in understanding the disease burden in middle-income scenarios. They provide valuable information on the incidence, risk factors, and outcomes of MI and can be used to identify disparities in the burden of disease and to monitor the effectiveness of prevention and treatment strategies. This information is essential for developing effective approaches to reducing the burden of MI in middle-income countries.
